# Supplementary material for: Effects of whole-body electromyostimulation on function, muscle mass, strength, social participation, and falls-efficacy in older people: A randomized trial protocol
Source: PLoS One. 2021 Jan 25;16(1):e0245809. doi: 10.1371/journal.pone.0245809 (PMC7833144; doi:10.1371/journal.pone.0245809)
Supplement: S2 Appendix — (DOCX) [file pone.0245809.s002.docx]

**TERMO DE CONSENTIMENTO LIVRE E ESCLARECIDO**

Gostaríamos de convidar você a participar como voluntário (a) da pesquisa “**Efetividade da estimulação elétrica muscular de corpo inteiro na estrutura, função muscular e risco de queda de idosos sedentários***”.* O motivo que nos leva a realizar esta pesquisa é conhecer se um programa de exercícios físico por meio eletroestimulação muscular produz os mesmos efeitos do que um programa de exercícios convencionais de levantar pesos em idosos que não praticam exercícios. Nesta pesquisa, os efeitos que vamos avaliar serão se a estimulação elétrica, comparado ao exercício de levantar peso, aumenta mais a força, o tamanho do seu músculo e o funcionamento destes músculos, e se esses efeitos irão reduzir o risco de você cair e melhorar sua independência nas atividades do dia-a-dia.

Deste modo, alguns estudos mostram que por meio da eletroestimulação muscular podemos ter uma melhora desses aspectos, com menos tempo de treinamento do que o treinamento com levantamento de pesos.

Ao concordar em participar dessa pesquisa, assinando abaixo, você deverá estar ciente de todas as etapas a que será submetido, conforme será explicado aqui.

Você irá passar inicialmente por uma avaliação cognitiva, na qual queremos avaliar seu grau de entendimento, são apenas poucas perguntas rápidas. Depois será feita a avaliação antropométrica, em que será medido seu peso e sua altura, e também fará um exame chamado de bioimpedância. Neste exame, a bioimpedância, serão posicionados quatro eletrodos adesivos nos braços e nas pernas, sem que ocorra choque ou qualquer outra situação desconfortável. Você não sentirá nada neste exame, ele é imperceptível, e através dele vamos medir e avaliar a quantidade de gordura e músculo que há em seu corpo. Você fará um teste para medir o quanto de força você tem para estender o seu joelho. Isso permite avaliar a força do músculo da coxa. Depois você será submetido a um teste de caminhar num corredor de 10 metros. Trata-se de um teste rápido e simples, no qual você deverá caminhar a maior velocidade possível no corredor (terreno plano). O objetivo desse teste é avaliar a velocidade em que você consegue caminhar em um percurso pequeno. Também faremos um teste de levantar e sentar na cadeira por 30 segundos, e um teste de levantar da cadeira, andar no plano por três metros, caminhar de volta e sentar na cadeira. Esses testes indicam como está sua capacidade de se movimentar nas situações do dia a dia. Antes e após a realização dos testes, iremos medir sua pressão arterial, sua frequência cardíaca, se sente falta de ar ou cansaço nas pernas - estes últimos por uma escala que vai de 0 a 10. Os testes realizados podem aumentar os batimentos do coração, aumentar sua pressão arterial, causar cansaço nas pernas e cansaço para respirar, mas estes são sinais normais durante algum esforço.

Estes sinais serão cuidadosamente monitorizados dentro de limites seguros, ou seja, o examinador poderá interromper os testes caso ameace a sua segurança durante o teste. Caso você não seja capaz de continuar o teste ou diante de qualquer desconforto, você poderá também pedir para parar o teste. Também será avaliado por meio de um questionário o seu medo de cair em situações como subindo ou descendo escadas, tomando banho, fazendo compras, etc. Também será avaliado o seu grau de independência para realizar atividades do cotidiano como tomar banho, trocar de roupa, se alimentar. Esses questionários para conhecer sua independência nos dia a dia e risco de queda, será avaliado antes do treinamento, depois de concluir o período de treinamento, e também após 3, 6 e 12 meses de terminado o treinamento por conversa telefônica. O objetivo desses questionários é saber se o treinamento dos músculos melhorou sua forma de realizar as tarefas do dia a dia e reduziu o risco de cair. Os riscos relacionados à aplicação desses questionários incluem exposição sobre a rotina, relembrar algumas sensações desgastantes tais como cansaço e medo de cair quando realiza algumas atividades, desconforto pelo tempo gasto no preenchimento do questionário, porém são riscos mínimos. Se isto ocorrer, você poderá interromper o preenchimento dos instrumentos a qualquer momento e também poderá desistir de participar da pesquisa. As entrevistas dos questionários serão realizadas em ambiente privativo, bem como haverá apenas a presença do pesquisador na sala individualizada para a coleta de dados.

Após a realização destas avaliações, será realizado um sorteio para indicar qual tipo de treinamento muscular você será submetido. Haverá um grupo que fará o treinamento muscular por meio da eletroestimulação de vários músculos: músculos das costas, abdômen, peitoral, anterior e posterior das coxas, anterior e posterior dos braços, e por isso chamamos essa técnica de estimulação elétrica muscular de corpo inteiro. Se neste grupo, você passará por um período de familiarização, onde você poderá se acostumar com a corrente elétrica gerada por uma vestimenta que contém eletrodos. As aplicações de eletroestimulação dão certo formigamento no início de cada aplicação, que vai ficando cada vez menor com o passar do tempo. Não há perigo de choque elétrico, pois os responsáveis pelo manuseio do aparelho são pessoas treinadas, e o aparelho é testado, calibrado e ajustado para usar com uma intensidade segura. Quando iniciar estudo você fará duas sessões de estimulação elétrica por semana, em dias diferentes, durante no máximo 20 minutos cada sessão. O outro grupo sorteado fará o treinamento muscular clássico de levantamento de pesos por cerca de 40 minutos, exercitando a força dos mesmos músculos relatados acima do grupo da eletroestimulação. Ambas as formas de treinamento (eletroestimulação ou levantamento de pesos) ocorrerão duas vezes por semana durante 8 semanas.

Independente, do grupo de treinamento que você irá participar, haverá um fisioterapeuta acompanhando todas as sessões de exercícios, e monitorizando seu cansaço, sua atividade e qualquer outra queixa que você tiver. Entretanto, é normal que o exercício provoque cansaço e aumento do batimento cardíaco, e você será orientado a manter um cansaço de nível moderado, o qual deve ser tolerável a você. E caso necessário, poderá pedir a qualquer momento para diminuir a intensidade do exercício. Qualquer outro sinal ou sintoma que represente risco para algum efeito indesejável do treinamento, este será interrompido e você será monitorado e colocado em repouso até sua pronta recuperação.

Essa pesquisa contribuirá para identificar se o exercício por eletroestimulação do corpo inteiro é mais eficaz do que o exercício de levantar pesos. Assim, sua participação nesta pesquisa poderá ajudar na escolha do tratamento para melhorar a força, a massa muscular e a capacidade física de idosos com perda muscular e com maior risco de quedas e maior dependência de outras pessoas, para que possa melhorar a qualidade de vida dessas pessoas.

A sua participação neste estudo é voluntária. Serão necessárias duas visitas por semana, num período de 10 semanas, ao laboratório de Avaliação do Desempenho Físico- Funcional da Faculdade de Fisioterapia da Universidade Federal de Juiz de Fora. É garantia de liberdade da retirada deste consentimento a qualquer momento deste programa, sem qualquer prejuízo a sua pessoa. Apesar disso, se você tiver algum dano por causadas atividades que fizermos com você nesta pesquisa, você tem direito a indenização. Você terá todas as informações que quiser sobre esta pesquisa e estará livre para participar ou recusar-se a participar. Mesmo que você queira participar agora, você pode voltar atrás ou parar de participar a qualquer momento. A sua participação é voluntária e o fato de não querer participar não vai trazer qualquer penalidade ou mudança na forma em que você é atendido (a). O pesquisador não vai divulgar seu nome. Os resultados da pesquisa estarão à sua disposição quando finalizada. Seu nome ou o material que indique sua participação não será liberado sem a sua permissão. Você não será identificado (a) em nenhuma publicação que possa resultar. As informações das suas avaliações serão analisadas e utilizadas para estudos/trabalhos realizados por alunos do mestrado em Ciências da Reabilitação sob supervisão, não sendo divulgada a identificação de nenhum paciente. Você receberá uma cópia dos resultados das suas avaliações e do seu treinamento no final da sua participação no estudo. Em qualquer etapa deste estudo você terá acesso aos profissionais responsáveis para o esclarecimento de suas dúvidas. Os principais responsáveis são os pesquisadores Carla Malaguti e Diogo Carvalho Felício, os quais podem ser encontrados nos telefones: 2102-3256 ou 99199-3329.

Este termo de consentimento encontra-se impresso em duas vias originais, sendo que uma será arquivada pelo pesquisador responsável e a outra será fornecida a você. Os dados coletados na pesquisa ficarão arquivados com o pesquisador responsável por um período de 5 (cinco) anos. Decorrido este tempo, o pesquisador avaliará os documentos para a sua destinação final, de acordo com a legislação vigente. Os pesquisadores tratarão a sua identidade com padrões profissionais de sigilo, atendendo a legislação brasileira (Resolução Nº 466/12 do Conselho Nacional de Saúde), utilizando as informações somente para os fins acadêmicos e científicos.

Declaro que concordo em participar da pesquisa e que me foi dada à oportunidade de ler e esclarecer as minhas dúvidas.

Juiz de Fora, de de 20 .

Assinatura do Participante Assinatura do (a) Pesquisador (a)

**Nome do Pesquisador Responsável: Carla Malaguti**

Faculdade de Fisioterapia

Departamento de Fisioterapia Cardiorrespiratória e Musculoesquelética – CEP: 36038-300 - Fone: 2102-3256 ou 99199-3329 -

E-mail: [carlamalaguti@gmail.com](mailto:carlamalaguti@gmail.com)
