# Supplementary material for: Effects of whole-body electromyostimulation on function, muscle mass, strength, social participation, and falls-efficacy in older people: A randomized trial protocol
Source: PLoS One. 2021 Jan 25;16(1):e0245809. doi: 10.1371/journal.pone.0245809 (PMC7833144; doi:10.1371/journal.pone.0245809)
Supplement: S1 File — (DOCX) [file pone.0245809.s003.docx]

| **Título do Projeto:** | **Efetividade da estimulação elétrica muscular de corpo inteiro na função, massa muscular, força, participação social e eficácia de quedas de idosos** |
| --- | --- |
| **Pesquisador Responsável:** | Carla Malaguti |
| **Equipe da Pesquisa:** | Cristino Carneiro Oliveira  Diogo Carvalho Felício  Diogo Simões Fonseca  Anderson José  João Luiz Quagliotti Durigan  José Elias Filho  Túlio Medina Dutra de Oliveira |
| **Endereços para contato:** | **E-mail:**carlamalaguti@gmail.com  **Tel.:**(32) 99199-3329 |
| **Unidade/Departamento**  **/Instituto/Instituição:** | Faculdade de Fisioterapia/ Departamento de Fisioterapia Cardiorrespiratória e Musculoesquelética/ Universidade Federal de Juiz de Fora. |

**Desenho:**

Este será um ensaio clínico aleatório a ser conduzido no Laboratório de Análise de Movimento da Faculdade de Fisioterapia da Universidade Federal de Juiz de Fora. Os participantes serão esclarecidos quanto aos objetivos da pesquisa e os que consentirem assinará o termo de consentimento Livre e Esclarecido. Mediante a aprovação do comitê de ética em pesquisa, este projeto será registrado na plataforma de Registros Brasileiros de Ensaios Clínicos (REBEC). Serão elegíveis para o estudo, idosos com idade superior a 60 anos, e ambos os sexos, sedentários, acompanhados no ambulatório de geriatria do hospital universitário da Universidade Federal de Juiz de Fora (HUCAS/UFJF), que serão convidados por meio de cartazes de divulgação do estudo ou por convite do pesquisador na sala de espera durante as visitas de consulta destes ao ambulatório. Após aplicados os critérios de elegibilidade para entrada no estudo, os participantes serão avaliados quanto aos testes funcionais: velocidade da marcha, teste de levanta-senta e o teste de TUG. Também serão avaliados quanto a composição corporal pela análise de quantidade de massa magra e massa gorda pela bioimpedância elétrica, força muscular de extensão do joelho, e entrevistas por questionários avaliando a participação social e o medo de cair. Após o período de intervenção de 8 semanas estes serão reavaliados em todas as avaliações, e depois seguidos por 3 e 6 meses com avaliações dos questionários de medo de cair e a participação social. Os participantes serão aleatoriamente alocados em algum braço do estudo, grupo de eletroestimulação ou grupo de treinamento resistido. **Grupo de eletroestimulação:** receberá treinamento baseado em eletroestimulação de corpo inteiro (ECI). Para definir os parâmetros da eletroestimulação serão utilizadas referências prévias que apresentaram resultados satisfatórios com a eletroestimulação de corpo inteiro em idosos (Kemmler et al, 2014, 2013). O grupo de eletroestimulação será submetido a 18 minutos de estimulação elétrica muscular, duas sessões semanais em dias alternados, durante 8 semanas. Será utilizada uma corrente elétrica bipolar intermitente com frequencia de 85 Hz, largura de pulso de 350 μs com 6 segundos de estimulação e 4 segundos de repouso. Serão estimulados as regiões de coxa, braço, abdômen, tórax, músculos paravertebrais cervicais e torácicos e grande dorsal. **Grupo de treinamento resistido:** realizará duas sessões semanais de treinamento com exercícios resistidos supervisionados, envolvendo os mesmos músculos do grupo de eletroestimulação, porém sem eletroestimulação, e com carga de 50 a 80% de uma repetição máxima, por um período de 8 semanas. Após a primeira reavaliação, os idosos serão acompanhados em intervalos de 3 e 6 meses por meio das avaliações dos questionários de participação social e medo de cair, a fim de avaliar se o efeito do treinamento se manteve ao longo de um ano.

**Resumo:**

É bem conhecido a elevada prevalência de sarcopenia em idosos e o papel do treinamento físico por meio de exercício resistido na sua reversão. No entanto, diversas são as barreiras e limitações que impedem a aderência de idosos a um programa de treinamento físico regular para aumentar a força ou massa muscular e reduzir o risco de quedas. Recentemente, a estimulação elétrica de corpo inteiro parece ser tempo efetiva na recuperação da sarcopenia com menos tempo de intervenção. Nesse sentido, este estudo objetiva avaliar a efetividade da eletroestimulação de corpo inteiro na funcionalidade, força e massa muscular de idosos, bem como no medo de cair e na participação social comparado ao treinamento resistido convencional. Serão envolvidos idosos sedentários, sem comprometimento cognitivo e neurológico, doenças crônicas agudizadas, e alterações musculoesqueléticas que impeçam a realização de exercício resistido. A amostra será aleatorizada em dois grupos. O grupo de eletroestimulação submetido a 18 minutos de estimulação elétrica muscular, duas sessões a por semana, alternadamente, durante oito semanas. Será utilizada uma corrente elétrica bipolar intermitente com frequência de 85 Hz, largura de pulso de 350 μs com 6 segundos de estimulação e 4 segundos de repouso. O grupo de treinamento resistido realizará exercícios com carga de 50 a 80% de uma repetição máxima em sessões supervisionadas, duas vezes por semana, num período de 8 semanas. A força muscular será avaliada por dinamômetro manual Microfet; a avaliação funcional será determinada pela velocidade da marcha, teste de TUG e teste levanta-senta, a massa muscular será avaliada por bioimpedância elétrica, além de questionários para avaliar o medo de cair e o grau de participação social. Os dados intergrupos serão comparados por teste-t pareado ou Mann-Whitney de acordo com a normalidade testada. Avaliações longitudinais após 3 e 6 meses de término do treinamento serão conduzidas por meio dos questionários de avaliação da participação social e do medo de cair. O tamanho de efeito será determinado para cada variável de interesse. Análise de modelos mistos, incluindo os desfechos medidos nos 4 momentos (basal, após intervenção, 3 e 6 meses) será utilizado. Espera-se que com os resultados deste estudo seja possível determinar a efetividade da estimulação elétrica de corpo inteiro na melhora da capacidade funcional de idosos e na redução do risco de quedas.

**Palavras-chave:**

Eletroestimulação muscular, Idoso, Sarcopenia.

**Introdução (revisão da literatura):**

O envelhecimento populacional é um proeminente fenômeno mundial. Os países em desenvolvimento vêm apresentando nas últimas décadas um progressivo declínio nas suas taxas de mortalidade e, mais recentemente, também nas suas taxas de fecundidade o que culmina com um aumento do contingente de idosos. A expressão definitiva desse envelhecimento pode ser observada na pirâmide populacional que se transforma, passando de um modelo de população em crescimento para um modelo de população estabilizada (Chaimowicz, 1997).

No Brasil, os dados sociodemográficos da Pesquisa Nacional por Amostra de Domicílios (PNAD) do Instituto Brasileiro de Geografia e Estatística (IBGE, 2012), constatou que em 1991 os idosos representavam 4,8% da população, em 2000 5,8%, e em 2010 chegam a 7,4%. Do total de 190.755.799 da população brasileira, 14.081.480 têm 65 anos ou mais. A estimativa do IBGE para o ano de 2025 equivale a 15% de idosos da população total, correspondendo aproximadamente a 30 milhões de idosos no país (IBGE, 2012). De acordo com a Organização Mundial de Saúde (OMS, 2005), até 2025 o Brasil será o sexto país do mundo em número de idosos.

No país, os avanços da medicina e as melhorias nas condições gerais de vida da população repercutem no sentido de elevar a expectativa de vida ao nascer de 45,5 anos de idade, em 1940, para 72,7 anos, em 2008, ou seja, mais 27,2 anos de vida. De acordo com a projeção do IBGE, o país continuará aumentando anos na vida média de sua população, alcançando em 2050 o patamar de 81,29 anos, basicamente o mesmo nível atual da Islândia (81,80), China (82,20) e Japão (82,60) (IBGE, 2012). O envelhecimento é acompanhado de um aumento na prevalência de doenças crônicas degenerativas e comorbidades, refletindo na diminuição da capacidade funcional, da qualidade de vida e da autonomia dos idosos (Bussche*et al.*, 2011). Dentre as alterações do processo de envelhecimento destaca-se a sarcopenia.

Em 1989, Rosenberg propôs o termo sarcopenia (do grego, *sark*= carne; *penia*= perda) para descrever a perda involuntária de massa muscular esquelética relacionada com a idade. Alguns autores consideram que além de haver perda de massa muscular, há também diminuição da força muscular (Sayer*et al*, 2008; Lang*et al*, 2010). Para outros autores, a sarcopenia é definida como perda de massa muscular esquelética, força muscular e limitação funcional (Evans, 1995; Roubenoff, 2001). Em 2010, na tentativa de elaborar uma definição operacional da sarcopenia, Cruz-Jentoft*et al* propuseram através de um consenso europeu que a sarcopenia é uma síndrome geriátrica caracterizada por perda de massa muscular e da função muscular (força ou performance física), sem a necessidade de doença para o seu aparecimento, embora o processo possa ser acelerado em decorrência de algumas doenças crônicas. De acordo com o consenso, a detecção de massa e força muscular pode ser detectada na prática clínica com estratégias mais acessíveis como dados antropométricos e força muscular de preensão palmar e na pesquisa científica com instrumentos mais fidedignos tais como a Tomografia Computadorizada e a dinamometria isocinética.

Depois dos trinta anos a massa muscular diminui aproximadamente 3-8% e essa taxa de declínio é mais acentuada após os sessenta anos (Melton*et al*, 2000). Baumgartner *et al* em 1998 realizaram um estudo epidemiológico para estimar a prevalência de sarcopenia em 883 idosos do Novo México. Verificaram um aumento na prevalência de sarcopenia de 13-24% em idosos acima de setenta anos para 50% em idosos acima dos oitenta anos. A sarcopenia foi significantemente associada com a inatividade física, morbidade, obesidade, renda e comportamentos de saúde. Iannuzzi-Sucich em 2002 em um estudo semelhante encontraram prevalência de 22,6% ao avaliar 195 mulheres de 64 a 93 anos e 26,8% ao avaliar 142 homens de 64 a 92 anos. Analisando mulheres e homens acima de 80 anos, os autores observaram que a prevalência passou para 31,0% e 52,9% respectivamente. Múltiplos fatores inter-relacionados contribuem para o desenvolvimento e progressão da sarcopenia. Com o envelhecimento postula-se que ocorra redução ou resistência às substâncias anabólicas no músculo esquelético como a diminuição do nível sérico de testosterona e androgênios, atrofia das fibras tipo II, declínio das unidades motoras, perda de motoneurônios alfa, ingestão reduzida de proteínas, redução do hormônio de crescimento, sedentarismo e imunosenescência (Roubenoff*et al*, 2000).

Dentre as modalidades terapêuticas de prevenção e tratamento da sarcopenia destaca-se o exercício resistido (Kryger, 2007).

Kruger e colaboradores avaliaram 6000 idosos e reportaram que apenas 11% estão inseridos em programas de treinamento resistido. Em outro estudo Sluijs e colaboradores propuseram um programa de exercícios resistidos para idosos com dor crônica e identificaram que 70% não obtiveram adesão satisfatória a longo prazo. Taylor e colaboradores acrescentaram que a taxa de adesão a exercícios em idosos é de aproximadamente de 14 a 17%. O termo adesão refere-se ao número de sessões realizadas dividido pelo número de sessões ofertadas (Hong SY et al, 2008). Dados da literatura sugerem que os resultados de uma intervenção com idosos são satisfatórios quando a adesão ao programa é em torno de 80-85% (Pistersetal, 2010).

Os profissionais de saúde devem realizar esforços para identificar os fatores relacionados a adesão a exercícios resistidos, no entanto, ainda não há um consenso na literatura sobre o tema. Idosos tendem a participar de forma mais assídua no início do programa e tendem a ser mais faltosos com o passar do tempo (Pisterset al, 2010). Nesse sentido, novas estratégias de treinamento podem ser uma opção para otimizar a adesão dos idosos a programas terapêuticos. Recentemente vem sendo empregado como uma alternativa a eletroestimulação do corpo inteiro (ECI).

A eletroestimulação do corpo inteiro é uma recente proposta para condicionamento muscular. Trata-se de uma técnica segura e não invasiva pelo equipamento mihabodytec® (Gersthofen, Germany) que permite a ativação simultânea de diversos grupamentos musculares. Uma das vantagens da ECI é que ela atua diretamente na síntese de proteína musculoesquelética com uma demanda de tempo bem menor do que as técnicas convencionais.

Estudos com idosos são incipientes. Em uma recente pesquisa, Kemmler e colaboradores (2014) realizaram um ensaio clínico aleatório o qual objetivaram avaliar a efetividade da ECI na composição corporal de idosos com risco de sarcopenia. Setenta e seis idosos foram alocados em dois grupos. O grupo experimental foi submetido a 18 minutos de eletroestimulação, três sessões a cada 14 dias totalizando 54 dias de intervenção. Ao término do programa os pesquisadores observaram resultados significativos do grupo experimental nas variáveis massa muscular apendicular, massa magra e força muscular de extensores de joelho e tronco.

Em outro estudo, foi investigado a efetividade da ECI na massa muscular apendicular e gordura abdominal de idosos. Quarenta e seis idosos sedentários foram alocados em grupo experimental (GE) e controle (GC). O GE (n=23) realizou 18 minutos de ECI, três sessões a cada 14 dias totalizando 54 dias de intervenção. Após 12 meses de intervenção foram encontrados resultados estatisticamente significativos a favor do GE nos desfechos de massa muscular apendicular e gordura abdominal (Kemmleret al 2013).

Apesar de apresentar resultados promissores, destaca-se que existem poucos grupos no mundo investigando a efetividade da ECI. Adicionalmente destaca-se que influências culturais e socioeconômicas podem mediar os resultados.

**Hipótese:**

A principal hipótese desse estudo é a de que a estimulação elétrica de corpo inteiro é tão efetiva em melhorar desfechos clínicos-funcionais em saúde quanto o treinamento resistido convencional.

**Objetivo primário**

O objetivo primário deste estudo é avaliar a efetividade da estimulação elétrica de corpo inteiro na capacidade funcional, força muscular, velocidade de marcha, risco de queda e massa magra de idosos sedentários quando comparada ao treinamento resistido convencional.

**Objetivo secundário**

Os objetivos secundários deste estudo é avaliar a efetividade da estimulação elétrica de corpo inteiro no medo de cair e na participação social de idosos sedentários quando comparada ao treinamento resistido convencional.

**Procedimentos e Instrumentos:**

Este será um ensaio clínico aleatório a ser conduzido no Laboratório de Análise de Movimento da Faculdade de Fisioterapia da Universidade Federal de Juiz de Fora. Os participantes serão recrutados por meio de cartazes na universidade, comunicações pessoais e divulgação pela internet e através de redes sociais. Os participantes serão esclarecidos e orientados quanto às avaliações e aos objetivos da pesquisa e os que consentirem assinarão o termo de consentimento Livre e Esclarecido (TCLE). As avaliações só terão início após os participantes assinarem o TCLE. Os participantes serão instruídos a utilizarem roupas confortáveis e calçados adequados e não realizar exercícios extenuantes pelo menos 24h antes da avaliação e também da sessão de exercícios. A alocação será randômica, realizada após a avaliação basal, por meio de uma tabela de números aleatórios para os grupos experimental e controle.

Todos os participantes serão submetidos às avaliações descritas abaixo no período basal, imediatamente após as oito semanas de intervenção, e também serão replicados os questionários de medo de cair e de avaliação da participação social após 3 e 6 meses depois do final do período de intervenção. Tanto a avaliação basal, quanto a reavaliação realizada após as oito semanas de intervenção, terão duração de aproximadamente 40 minutos. Após a primeira reavaliação, os idosos serão acompanhados em intervalos de 3 e 6 meses através dos questionários de participação social e medo de cair, por meio de ligações telefônicas, a fim de avaliar se o efeito do treinamento se manteve ao longo de um ano. A ligação para responder o questionário durará no máximo 15 minutos, que é a média de tempo de reposta para os dois questionários (Tabela 2).

Triagem: será utilizado o Instrumento de Triagem (ANEXO 1) para coletar dados sobre características demográficas (por exemplo, idade, sexo) e clínicas (medicações, co-morbidades, antecedentes clínicos e história familiar).

O Six-Item Screener (SIS) (Anexo II) é um instrumento simples desenvolvido por Callahanet al. (2001), derivado do *Mini-mental State Examination,* podendo ser administrado por telefone ou sob forma de entrevista, visando identificar comprometimento cognitivo. Seu escore varia entre zero e seis, é facilmente marcado por uma soma simples de erros e leva em conta a orientação temporal (três itens) e recordação tardia (três itens). O SIS será aplicado logo após o Instrumento de Triagem e aqueles pacientes que obtiveram uma pontuação menor que quatro, serão considerados com comprometimento cognitivo sendo assim, excluídos do estudo.

**Avaliação física funcional**

Será avaliada a **velocidade habitual da marcha** (Anexo III). Para avaliação da velocidade de marcha habitual será utilizada a relação distância/tempo (m/s), medida em um espaço de 10 metros. Os participantes serão instruídos a andar em velocidade auto-selecionada. A velocidade da marcha será registrada apenas nos 6 metros centrais da pista, identificados lateralmente por marcas de fita, para evitar viés de aceleração e desaceleração. Os participantes serão instruídos a permanecer em pé com os dois pés atrás da linha de início e iniciar a marcha após o comando verbal “*ande no seu ritmo normal até a última marca no chão, ou seja, como se estivesse andando na rua para fazer uma compra na padaria”*; (Fritz 2009).

Outra avaliação funcional é o teste de **The timed Up &Go** (TUG) que será realizado de acordo com as recomendações (Anexo IV). Os participantes estarão sentados em uma cadeiras em braços e serão solicitados a levantar e caminhar três metros, virarem um cone, caminhar de volta e sentar na cadeira. Os participantes serão incentivados à realizar esse percurso o mais rápido possível sem correr. O desfecho é o tempo gasto para completar a tarefa (Podsiadlo et al. 2009).

Adicionalmente, o **teste levanta-senta** que será realizado usando uma cadeira sem braços, com uma cadeira com altura de 43,2 cm (Anexo V). A cadeira será colocada contra a parede para prevenir o deslocamento desta durante o teste. O teste inicia com o participante sentado no meio da cadeira, costas retas, pés aproximadamente na largura dos ombros e apoiados no chão em um ângulo ligeiramente atrás dos joelhos, com um pé ligeiramente à frente do outro para ajudar a manter o equilíbrio quando em pé. Os braços serão cruzados contra o peito. No sinal de "vai", o participante deve se levantar (corpo ereto e reto) e depois voltar para a posição sentada inicial. Os participantes serão incentivados a realizar tantos ciclos completos de sentar-levantar quanto possível dentro de um limite de tempo de 30 segundos (Joneset al. 1999).

**Avaliação da força muscular de extensão de joelho:** medidas da força do músculo quadríceps por meio da extensão do joelho esquerdo e direito será avaliada utilizando o dinamômetro manual Microfet (DMM) por meio de testes isométricos (Anexo VI). O DMM é uma ferramenta de avaliação confiável e válida para medir a força de extensão de joelho em idosos (Arnold 2010). Com o participante sentado em uma cadeira alta, com os pés sem tocar o chão, as pernas em posição vertical, e o dinamômetro aplicado perpendicular à perna apenas proximal aos maléolos (Figura 1), e fixado por uma cinta a cadeira e a determinação da angulação será realizada com goniômetro. O participante deverá fazer força para estender o joelho contra o dinamômetro manual. Durante a avaliação será dado o estímulo verbal: “*estique o joelho fazendo sua maior força, estique, estique, estique”*. Essa medida será realizada cinco vezes, sendo descartados o maior e o menor valor de força registrado. A média dos três valores restantes será calculada, e os valores de força muscular isométrica de extensão do joelho serão registrados em Newton.


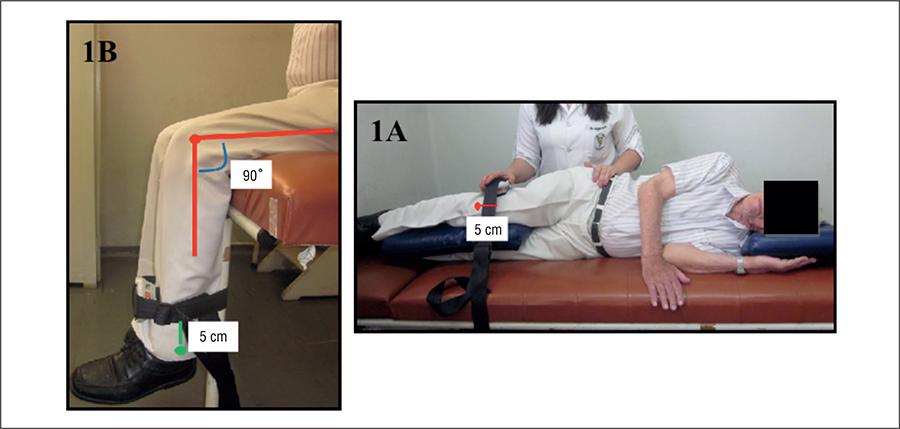


Figura 1. Posicionamento do paciente para a avaliação da força extensora do joelho.

**Avaliação da massa muscular:** a análise da composição corporal será medida pelo aparelho de bioimpedância elétrica *Biodynamics* tetrapolar, comumente descrito na literatura, com apresentação digital, fornecendo os valores de resistência e reactância. O exame baseia-se na passagem de uma baixa corrente elétrica (500 a 800 μA e 50 kHz) através do corpo, esta imperceptível ao participante avaliado (Anexo VII).

Antes da realização do exame serão verificadas as orientações prévias dadas aos participantes. As orientações requerem ao paciente que compareçam ao local do exame em jejum; que o mesmo não realize atividade física até 12 horas antes; não faça uso de bebida alcoólica por 48 horas e que esvazie a bexiga 30 minutos antes das medidas. As medidas de bioimpedância serão obtidas do lado direito do paciente, que será orientado a manter-se em posição supina (deitada), retirando os calçados e meias, tendo o cuidado de manter os membros superiores e os inferiores separados do corpo em aproximadamente 30°, a partir de uma linha média. Os pontos corporais que tiverem contato com os eletrodos serão limpos com álcool, a fim de se retirar a oleosidade da pele e garantir melhor contato com o eletrodo. Os eletrodos serão descartados após cada medição. Os cabos pretos (injetores) serão conectados nos eletrodos dos pés e os cabos vermelhos (receptores) nos eletrodos das mãos. As pinças de conexão “jacarés” serão conectadas nos eletrodos distais, isto é, sobre o dedo médio da mão ou próximo aos dedos do pé. As pinças de cor vermelha serão colocadas nos eletrodos proximais, isto é, aqueles colocados no punho e no tornozelo. O eletrodo de detecção (pinça vermelha) será colocado no pulso, em uma linha imaginária que divide a ulna e inicia-se na protuberância óssea do punho; o eletrodo de pinça preta será colocado no dedo médio. No pé, serão colocados os eletrodos em uma linha imaginária que divide os maléolos medial e lateral utilizando-se a pinça vermelha. A pinça preta será colocada acima dos nós dos dedos do pé (Figura 2).

A impedância, em oposição ao fluxo da água corrente é então, medida. Antes da realização do exame, será verificado se as orientações dadas no momento da entrevista foram cumpridas. As medidas serão tomadas na posição supina após 15 minutos de repouso. Por meio dos valores obtidos de resistência e reactância, pode ser calculado os valores de massa magra (kg), pela equação: (MM = água corporal total/ 0,732; sendo: água corporal total = 0,69 x altura^2^ x resistência + 0,8. Com o valor obtido de massa magra (kg), será calculado o IMM – índice de massa magra, analogia feita ao IMC, utilizando-se a relação massa magra (em quilograma)/estatura^2^ (em metros), para a determinação da depleção muscular (Pichard*et al*.,2000).

Figura 2. Ilustração do posicionamento dos eletrodos durante a avaliação de bioimpedância.

**Avaliação da massa corporal e da altura:** A medida da altura do voluntário, expressa em centímetros (cm), será realizada em posição ortostática e após uma inspiração profunda, mantendo os pés unidos e com o peso do corpo distribuído entre eles. A estatura será tomada por estadiômetro e os valores serão aproximados em 0,5 cm. A massa corporal será aferida em uma balança mecânica antropométrica *Welmy®.* Esta será realizada com os pacientes descalços e com vestes leve, e expressa em quilograma (Kg).

**Participação social**: será avaliada pelo *Assessment of Life Habits* (*LIFE-H*)^21,22^, o qual é dividido em duas subescalas (“Atividades diárias” e “Papéis sociais”) que incluem doze domínios relacionados a nutrição, condicionamento físico, cuidados pessoais, comunicação, moradia e mobilidade, responsabilidades, relacionamentos interpessoais, vida em comunidade, educação, trabalho e recreação. Essas categorias correspondem aos capítulos do componente de atividade e participação da CIF. Cada item é pontuado de acordo com o nível de realização e o tipo de assistência necessária para realizar cada atividade, as pontuações podem variar de zero (restrição total na participação) a nove (máxima participação). Para se ter a pontuação de cada domínio utiliza a seguinte fórmula: (Σ pontuações*10)/(número de itens aplicáveis*9), e para pontuação total da escala é necessário fazer a média da pontuação dos domínios considerados. No presente estudo, devido à sua irrelevância para a maioria dos idosos, as categorias “emprego” e “educação” serão removidas da análise, restando 10 categorias e 59 itens.

**Avaliação do medo de cair:** será avaliado pela Escala de Eficácia de Quedas Internacional-Brasil (FES-I-BRASIL)(Anexo IX).A FES-I-Brasil é uma escala adaptada e validada para a população brasileira que apresenta adequada confiabilidade intra e interavaliadores. Este questionário será aplicado pelo fisioterapeuta pesquisador, treinado a essa avaliação, e que será realizada por meio de entrevista em ambiente privado e reservado. É composta por 16 atividades diárias variadas, desde atividades mais simples, como o banho, até atividades que exigem maior grau de independência, como a participação em atividades sociais (Camargos et al, 2007). Cada item de resposta vai de 1 a 4 pontos. Os escores ≥ 23 foram identificados como associação ao histórico de queda esporádica e ≥ 31 pontos, com associação à queda recorrente.

**Intervenção:** Após a avaliação basal e a aleatorização, os participantes serão submetidos a uma das duas modalidades de treinamento muscular (eletroestimulação ou resistido) por um período de oito semanas, como descritos abaixo. Em qualquer uma da modalidade de treinamento (eletroestimulação ou resistido) será permitido até no máximo dois participantes concomitantemente treinando em cada modalidade de treinamento. Independente da modalidade de treinamento será oferecido dois períodos semanais para cada participante treinar: terças e quintas-feiras ou segundas e quartas-feiras nos períodos da manhã ou tarde.

**Grupo de eletroestimulação:** os participantes alocados nesse grupo serão submetidos a eletroestimulação de corpo inteiro, a qual pode ativar simultaneamente de 8 a 12 grupos musculares (parte superior das pernas, parte superior dos braços, abdome, tórax, parte inferior das costas, parte superior das costas e grande dorsal) com diferentes ajustes de intensidade. Este treinamento será realizado no laboratório de Desempenho Físico-Funcional da Faculdade de Fisioterapia da UFJF, com até no máximo dois participantes concomitantemente por sessão. A corrente elétrica bipolar por dispositivos da MihaBodytec® (Gersthofen, Alemanha), será inicialmente aplicada com os seguintes parâmetros: freqüênciade 85Hz, amplitude de pulso de 350𝜇s, intermitentemente com 6 s de estimulação para realizar o movimento e 4 s de repouso (Tabela 1). A intensidade da corrente pode ser individualmente selecionada e modificada durante a sessão de eletroestimulação. O protocolo aplicado será de movimento intermitente de baixa intensidade/baixa amplitude baseado em parâmetros descritos em outros estudos (Kemmler et al, 2014, 2013). Os participantes serão conduzidos e supervisionados durante 18 minutos de treinamento de eletroestimulação de corpo inteiro, duas vezes por semana, sempre alternadamente, durante 8 semanas. As sessões de treinamento serão monitorizadas por um fisioterapeuta treinado, e também com estímulos visual e acústico guiados por vídeos que imitam exatamente os movimentos do protocolo. Com base em outros estudos, o protocolo de ECI seguirá as configurações comerciais do ECI com a estratégia de baixa carga e baixa amplitude de movimento. Envolvendo cinco movimentos básicos (“exercícios centrais”) dados na Tabela 1, dos quais serão combinados e levemente modificados para gerar 12 exercícios dinâmicos que serão realizados sem qualquer carga adicional em uma posição em pé. O treinamento com a ECI será estruturado em uma a duas séries de 6 a 8 repetições. A amplitude, a velocidade e a intensidade correspondente geradas pelo movimento serão baixas (isto é, semiagachamento: flexão da perna<35°) para evitar efeitos do próprio exercício, mas serão importantes para ajudar na ativação muscular. Além disso, nenhum incremento progressivo relacionado aos exercícios será aplicado durante o estudo. Após o período de adaptação de 4 sessões de ECI, a intensidade da corrente será ajustada individualmente de acordo com a tolerância dos participantes. Considerando a dificuldade da precisão na prescrição da intensidade da eletroestimulação devido às diferenças regionais, os participantes serão orientados a manter uma taxa de esforço percebido de “forte” (Escala entre “5 a 7” de 0-10) (Borg, 1990) durante a contração. A intensidade da corrente é o elemento chave para os efeitos positivos quando comparado aos programas de exercícios convencionais. A intensidade da corrente correspondente para cada região do corpo será salva no chip do equipamento para gerar uma configuração rápida, confiável e válida durante as sessões subseqüentes do ECI.

Tabela 1.Movimentosconcomitantesa ECI.

1. Semi-agachamento (6 sdown) com extensão de braço / levantamento do peso (6 s para cima) com flexão de braço

2. Semi-gachamento (6 s para baixo) com flexão do tronco (abdominais)

3. Semi-agachamento (6 s para baixo) com polias/ semi-agachamento (6 s para cima) com levantamento

4. Semi-agachamento (6 s para baixo) com abdução de braços (costas) / semi-agachamento (6 s para cima) com adução de braços (peitoral)

5. Semi-agachamento (6 s para baixo) e com exercício de remadaalta (6s para cima)

**Grupo de treinamento resistido:** os participantes desse grupo receberam o treinamento físico na sala de musculação da Faculdade de Educação Física da UFJF. Cada sessão de treinamento incluirá um aquecimento de 10 minutos, incluindo uma caminhada e movimento de diferentes segmentos corporais: braços, punhos, dedos, ombros, pernas e tornozelos. O treinamento resistido envolverá oito semanas em aparelhos de musculação (puxador frente, extensor de joelho, supino reto, puchador costas, etc.) envolvendo todos os maiores grupos musculares. Os participantes deste grupo de treinamento resistido deverão frequentar duas sessões por semana, durante um período de 50 a 60 minutos cada. Adaptações individuais do protocolo de treinamento serão feitas regularmente em função do desempenho individual.Para definir a intensidade de treinamento será estimada a carga de 1 RM. A 1RM tem sido amplamente utilizado como padrão ouro para a verificação da força muscular. Na quantificação da 1 RM, será a realização de tentativas repetidas para obtenção de cargas similares ao teste de 1RM (três a cinco tentativas) e também o mesmo intervalo entre as tentativas (dois a cinco minutos). A maior carga executada pelo participante, bem posicionado e sem compensação do movimento, será definida como a 1 RM. A intensidade será baseada no número de repetições possíveis (semana 1-2: quinze repetições, semana 2-6: nove repetições, com intensidade de 50-60% 1RM nas duas primeiras semanas, e depois com 70-80% 1RM. Os participantes serão continuamente questionados quanto a percepção de esforço por meio da escala de percepção de sintomas de Borg. Em caso de extremo cansaço, a intensidade poderá ser reduzida ou a sessão poderá ser interrompida. Será oferecido um volume de treinamento de três séries por exercício e 4,0 segundos entre as repetições e 60 segundos de descanso entre as séries, e os participantes serão estimulados a manter a contração muscular durante 6 segundos. Poderão ser treinados simultaneamente até dois participantes por sesssão. Este treinamento será rigorosamente acompanhado por um fisioterapeuta e um educador físico experiente e treinado para o protocolo de estudo.

A duração das sessões das duas intervenções são diferentes (18 minutos para o grupo de eletroestimulação *vs* 50-60 minutos para o grupo de treinamento resistido), em função que o treinamento de eletroestimulação de corpo inteiro estimula a contração muscular simultânea de vários grupos musculares sendo, portanto, tempo-efetiva em comparação ao treinamento resistido que trabalha isoladamente cada grupo muscular selecionado.

Tabela 2. Descrição das fases do estudo.


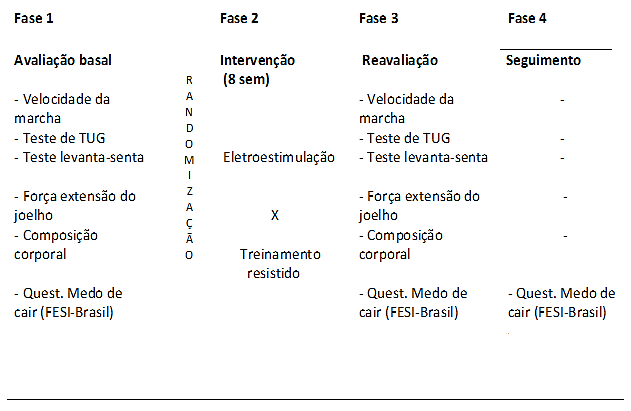


**(3 e 6 meses)**

- Quest. Participação social (Life-H)

- Quest. Participação social (Life-H)

- Quest. Participação social (Life-H)

**Critérios de inclusão:**

Serão incluídos no estudo uma amostra de conveniência de idosos com idade superior a 60 anos e sedentários, de ambos os sexos, com nível cognitivo que permita a compreensão das avaliações e intervenções do estudo, recrutados do ambulatório de geriatria do Hospital Universitário da Universidade Federal de Juiz de Fora (HUCAS/UFJF), convidados por meio de cartazes de divulgação do estudo e/ou por convite do pesquisador na sala de espera durante as visitas de consulta destes ao ambulatório.

**Critérios de exclusão:**

Serão excluídos idosos que apresentarem alterações cognitivas detectáveis pelo ponto de corte igual ou inferior a quatro no Six Item Screener (ANEXO I) Callahanet al. (2001), doença inflamatória na fase aguda, doenças cardiovasculares e metabólicas agudizadas, neoplasia em atividade nos cinco anos anteriores, uso de medicamentos anti-inflamatório, presença de sequelas neurológicas, fraturas ou osteossínteses cirúrgicas nos últimos seis meses e dificuldade visual e auditiva graves. Essas doenças ou comorbidades serão pesquisadas em prontuário do paciente no ambulatório de geriatria no qual é acompanhado e investigados pelo instrumento de triagem.

**Riscos:**

Os testes realizados como velocidade da marcha ao caminhar, levantar e sentar da cadeira pode aumentar a frequência cardíaca, e a resposta pressórica arterial, causar fadiga e dispnéia leve a moderada, mas estes são sinais normais durante o esforço submáximo. Estes sinais serão cuidadosamente monitorizados dentro de limites seguros, ou seja, o examinador poderá interromper os testes caso ameace a segurança do participante durante cada teste. Caso o participante não seja capaz de continuar o teste ou diante de qualquer desconforto extremo, este poderá também pedir para parar o teste.

O grupo de idosos que realizará o treinamento muscular resistido com levantamento de pesos poderá sentir fadiga muscular ao final da série de exercícios, entretanto, essa sensação será continuamente monitorizada por um fisioterapeuta treinado e experiente, e que permitirá a interrupção caso a fadiga seja intensa. Durante o treinamento resistido poderá haver elevação discreta da pressão arterial, da freqüência cardíaca, e da freqüência respiratória, sinais normais de percepção de esforço físico moderado, entretanto, estes sinais devem retornar aos valores basais após alguns segundos de término do esforço. Qualquer outro sinal ou sintoma que represente risco para algum efeito adverso do treinamento, este será interrompido e o participante será monitorado e colocado em repouso até sua pronta recuperação.

A eletroestimulação pode dar uma sensação de discreta de formigamento durante sua utilização, porém sem maior desconforto. Em caso de cãibras ou dores musculares essa será imediatamente interrompida. Dessa forma, os riscos são mínimos. Os participantes serão assistidos por profissionais e pesquisadores experientes e treinados para evitar qualquer intercorrência associada aos procedimentos do estudo. Em caso do participante não alcançar a pronta recuperação o SAMU poderá ser acionado.

Os riscos relacionados à aplicação dos questionários do projeto de pesquisa incluem exposição sobre a rotina, relembrar algumas sensações desgastantes tais como cansaço e medo de cair quando realiza algumas atividades, desconforto pelo tempo gasto no preenchimento do questionário, são mínimos, porém potenciais. Se alguma dessas situações posteriormente citadas ocorrer, você poderá interromper o preenchimento dos instrumentos a qualquer momento e também poderá desistir de participar da pesquisa. As entrevistas dos questionários serão realizadas em ambiente privativo, bem como haverá apenas a do pesquisador na sala individualizada para a coleta de dados. Os pesquisadores também garantem o sigilo e anonimato dos participantes. Os dados serão apresentados apenas em médias do grupo estudado. Nenhum dado individual será exposto.

**Benefícios:**

Esse estudo contribuirá para identificar se o exercício por eletroestimulação do corpo inteiro é mais eficaz do que o exercício resistido (de levantar pesos). Assim, a participação do voluntário nesta pesquisa poderá vir a ajudar na escolha do tratamento para melhorar a força, a massa muscular e a capacidade física de idosos com sarcopenia e com maior risco de quedas e incapacidade física, para que assim se possa melhorar a qualidade de vida dessas pessoas. A vantagem desse estudo é que se comprovada a eficácia da eletroestimulação sobre o treinamento resistido, essa traz economia de tempo no processo de treinamento físico, pois a eletroestimulação demanda menor tempo de treinamento físico do que o treinamento resistido. Sendo assim, essa forma de treinamento pode proporcionar maior tolerância e aderência em idosos sarcopênicos.

**Metodologia de análise de dados:**

**Análise estatística**

Será utilizado o SPSS versão 20.0. Os dados serão analisados em média e DP ou mediana e interquartil. Análise estatística por intenção de tratar será realizada por test t não pareado ou Mann-Whitney. O tamanho de efeito será determinado para cada variável de interesse. Análise de modelos mistos, incluindo os desfechos medidos nos 4 momentos (basal, após intervenção, 3 e 6 meses) será utilizado. O “momento” será incluído como uma variável de classe.  As interações grupo, tempo e grupo * tempo serão inseridas. Este termo de interação avalia o efeito puro da intervenção, uma vez que é a diferença, em cada ponto no tempo, entre as alterações da linha de base no grupo de estimulação de corpo inteiro e no grupode treinamento resistido. Um valor de p =0,05 será assumido para todos os testes.

**Desfecho Primário:**

Mudança na força muscular de extensão do joelho, e na funcionalidade da velocidade da marcha e número de repetições no teste senta-levanta, bem como a mudança em quilograma da massa magra.

**Desfecho Secundário:**

Mudanças na eficácia de quedas avaliada pelo FES-I/Brasil e participação social pelo Life-H.

**Tamanho da Amostra no Brasil:**

A previsão é de que sejam envolvidos 66 idosos no estudo, sendo 33 para cada braço do estudo. Entretanto, para se conhecer o tamanho do efeito um estudo piloto com 10 idosos inicialmente será realizado para fornecer subsídios para confirmar a previsão baseado no cálculo da amostra.

**Haverá uso de fontes secundárias de dados (prontuários, radiografias, dados demográficos, etc.)?**

( ) Sim ( X) Não

| **Informe o número de indivíduos abordados pessoalmente, recrutados, ou que sofrerão algum tipo de intervenção:**  **66** | | | **Número:**  **66** |
| --- | --- | --- | --- |
| **Grupos em que serão divididos os participantes da pesquisa** | | | |
| **Grupo** | **Nº de participantes** | **Intervenções a serem realizadas** | |
| Grupo  Elestroestimulação | 33 | Avaliação funcional: velocidade da marcha, teste de TUG e teste levanta-senta. Avaliação da força muscular de extensão do joelho. Avaliação da massa magra corporal. Avaliação da participação social e avaliação do medo de cair. Treinamento muscular por eletroestimulação de corpo inteiro. | |
| Grupo  Treinamento Resistido | 33 | Avaliação funcional: velocidade da marcha, teste de TUG e teste levanta-senta. Avaliação da força muscular de extensão do joelho. Avaliação da massa magra corporal. Participação social e avaliação do medo de cair. Treinamento muscular por meio de exercícios resistidos. | |

**Cronograma de Execução:**

A coleta de dados iniciará apenas após a aprovação do projeto pelo CEP.

| **Identificação da Etapa** | **Início (DD/MM/AAAA)** | **Término (DD/MM/AAAA)** |
| --- | --- | --- |
| Treinamento da equipe aos procedimentos do estudo | 02/04/2020 | 01/05/2020 |
| Coleta de dados | 01/06/2020 | 08/06/2021 |
| Análise dos dados | 10/06/2021 | 02/11/2021 |
| Triagem de participantes | 01/05/2020 | 02/11/2020 |
| Redação do relatório final e do manuscrito | 21/02/2022 | 25/02/2022 |
| Descrição dos resultados | 19/11/2021 | 22/02/2022 |

**Orçamento financeiro:**

Material a ser adquirido com recursos próprios do pesquisador:

A instrumentação de pesquisa necessária será adquirida mediante a liberação dos recursos do projeto aprovado no Edital Universal FAPEMIG APQ-03054-17:

| **Identificação do item** | **Quantidade** | **Capital**  **Valor em Reais (R$)** |
| --- | --- | --- |
| 01 | **Eletroestimulador de corpo inteiro**  **Marca:**mihabodytec® **Função:** ativação simultânea de grupamentos musculares | 47.000,00 |

Instrumentação disponível no Laboratório de Avaliação de Desempenho Físico-Funcional da Faculdade de Fisioterapia da UFJF:

| **Identificação do item** | **Quantidade** | **Capital**  **Valor em Reais (R$)** |
| --- | --- | --- |
| 01 | **Dinamômetro manual**  **Marca:**Mircrofet**Função:** avaliação da força muscular | 3.000,00 |
| 01 | **Bioimpedância elétrica**  **QUANTUM II - Marca**: RJL Systems, Inc. (USA) **Função**: avaliação da composição corporal | 11.800,00 |

**Financiamento:**

A instrumentação de pesquisa necessária será adquirida mediante a liberação dos recursos do projeto aprovado no Edital Universal FAPEMIG APQ-03054-17, a saber, o eletroestimulador de corpo inteiro. Os demais equipamentos necessários à realização do projeto já foram adquiridos pelo pesquisador em editais anteriores e encontram-se disponíveis no Laboratório de Avaliação de Desempenho Físico-Funcional da Faculdade de Fisioterapia da UFJF. Outras demandas menores como cartucho e papel para impressão dos protocolos serão realizados com recursos próprios do pesquisador.

**Propõe dispensa do TCLE:**

( ) Sim ( X ) Não

**Haverá retenção de amostras para armazenamento em biobanco/biorrepositório?**

( ) Sim ( X ) Não

**Referências:**

ARNOLD CM, WARKENTIN KD, CHILIBECK PD, MAGNUS CR. The reliability and validity of handheld dynamometry for the measurement of lower-extremity muscle strength in older adults. J Strength Cond Res 2010;24:815-24.

ASSUMPÇÃO FSN, FARIA-FORTINI I, BASÍLIO ML, MAGALHÃES LC, CARVALHO AC, TEIXEIRA-SALMELA LF. Adaptação transcultural do LIFE-H 3.1: um instrumento de avaliação da participação social. Cad Saúde Pub 2016: 32

BAUMGARTNER RN, KOEHLER KM, GALLAGHER D et al. Epidemiology of sarcopenia among the elderly in New Mexico. Am J Epidemiol 1998; 147:755-63.

BORG G. Psychophysicalscalingwithapplications in physicalworkandtheperceptionofexertion. Scand J WorkEnviron Health1990; 16 Suppl1:55-8.

BUSSCHE HVD, KOLLER D, KOLONKO T. et al. Which chronic diseases and disease combinations are specific to multimorbidity in the elderly? Results of a claims data based cross-sectional study in Germany. BMC Public Health 2011;11:1-9.

CAMARGOS FF, DIAS RC, DIAS JM, FREIRE MT. Cross-cultural adaptation and evaluation of the psychometric properties of the Falls Efficacy Scale-International Among Elderly Brazilians (FES-I-BRAZIL). Rev Bras Fisioter. 2010;14:237-43.

CHAIMOWICZ F. A saúde dos idosos brasileiros às vésperas do século XXI: problemas, projeções e alternativas. Rev Saúde Pública1997;31:184-200.

CRUZ-JENTOFT AJ, BAEYENS JP, BAUER JM et al. Sarcopenia: European consensus on definition and diagnosis. Age and Ageing, 2010:39; 412-23.

EVANS, WJ. What is sarcopenia? J Gerontol A BiolSci Med Sci 1995;50:50-55.

FRITZ S, LUSADDI M. White paper: walking speed: the sixth vital sign. J Geriatr Phys Ther2009; 32:2-5.

HONG SY, HUGHES S, PROHASKA T. Factors affecting exercise attendance and completion in sedentary older adults: a meta-analytic approach. J Phys Act Health. 2008;5:385–397.

IANNUZZI-SUCICH, M.; PRESTWOOD, K. M.; KENNY, A. M. Prevalence of sarcopenia and predictors of skeletal muscle mass in healthy, older men and women. J Gerontol A BiolSci Med Sci, 2002;57:772–77.

INSTITUTO BRASILEIRO DE GEOGRAFIA E ESTATÍSTICA (IBGE). Censo Demográfico. Ministério do Planejamento e Orçamento, https://agenciadenoticias.ibge.gov.br/agencia-noticias/2012-agencia-de-noticias/noticias/20980-numero-de-idosos-cresce-18-em-5-anos-e-ultrapassa-30-milhoes-em-2017

JONES CJ, RIKLI RE,BEAM WC. A 30-s Chair-Stand Test as a MeasureofLowerBodyStrength in Community-ResidingOlderAdults. ResearchQuarterly for Exerciseand Sport. 1999; 79:113-119.

KEMMLER W, BEBENEK M, ENGELKE K, VON STENGEL S. Impact of whole-body electromyostimulation on body composition in elderly women at risk for sarcopenia: the Training and ElectroStimulation Trial (TEST-III). Age (Dordr). 2014;36:395-406.

KEMMLER W, VON STENGEL S. Whole-body electromyostimulation as a means to impact muscle mass and abdominal body fat in lean, sedentary, older female adults: subanalysis of the TEST-III trial. ClinInterv Aging. 2013;8:1353-64.

KRUGER J, BROWN DR, GALUSKA DA, BUCHNER D. Strength training among adults aged $65 years – United States, 2001. MMWR Morb Mortal Wkly Rep. 2004;53:25–28.

KRYGER AI, ANDERSEN JL. Resistance training in the oldest old: consequences for muscle strength, fiber types, fiber size, and MHC isoforms. Scand J Med Sci Sports 2007;17:422-30.

LANG T, STREEPER T, CAWTHON P,BALDWIN K, TAAFFE DR, HARRIS TB. Sarcopenia: etiology, clinical consequences, intervention and assessment. OsteoporosInt 2010;21:543-59.

NOREAU L, DESROSIERS J, ROBICHAUD L, FOUGEYROLLAS P, ROCHETTE AM, VOSCOGLIOSI C. Measuring social participation: reliability of the LIFE-H in older adults with disabilities. Disab and Rehab 2004; 26:346–352

OMS, Organização Mundial de Saúde. Envelhecimento ativo: uma política de saúde / OMS; tradução Suzana Gontijo. – Brasília: Organização Pan-Americana da Saúde, 2005.

PICHARD C, KYLE UG, BRACCO D, SLOSMAN DO, MORABIA A AND SCHUTZ Y. Reference values of fat-free and fat masses by bioelectrical impedance analysis in 3393 healthy subjects. Nutrition 2000; 16:245-54.

PISTERS MF, VEENHOF C, SCHELLEVIS FG, TWISK JW, DEKKER J, DE BAKKER DH. Exercise adherence improving long term patient outcome in patients with osteoarthritis of the hip and/or knee. Arthritis Care Res. 2010;62:1087–1094.

PODSIADLO D, RICHARDSON S. The timed "Up & Go": a test ofbasicfunctionalmobility for frailelderlypersons. J AmGeriatr Soc. 1991;39:142-8.

RAICHE M, HEBERT R, DUBOIS MF, GUEYE NDR, DUBUC N. Yearly transitions of disability profiles in older people living at home.EurGeriatr Med2012 55:399-405

ROUBENOFF R, CASTANEDA C. Sarcopenia: understanding the dynamics of aging muscle. J Am Med Assoc 2001;286:1230-1.

SAYER, A. A.; COOPER, C.; EVANS J. R. et al. The development origins of sarcopenia: using peripheral quantitative computed tomography to assess muscle size in older people. J Gerontol A BiolSci Med Sci, 2008;63:835-840.

SLUIJS EM, KOK GJ, VAN DER ZEE J. Correlates of exercise compliance in physical therapy. Phys Ther. 1993;73:771–782.

TAYLOR WC, SALLIS JF, LEES E, et al. Changing social and built environments to promote physical activity: Recommendations from low income, urban women. Journal of Physical Activity and Health. 2007;4:54–65.
