# Supplementary material for: Effects of whole-body electromyostimulation on function, muscle mass, strength, social participation, and falls-efficacy in older people: A randomized trial protocol
Source: PLoS One. 2021 Jan 25;16(1):e0245809. doi: 10.1371/journal.pone.0245809 (PMC7833144; doi:10.1371/journal.pone.0245809)
Supplement: S2 File — (DOCX) [file pone.0245809.s004.docx]

| **Project Title:** | **Effectiveness of whole-body electrical muscle stimulation on the function, muscle mass and strength, social participation, and falls-efficacy in the elderly** |
| --- | --- |
| **Responsible Researcher:** | Carla Malaguti |
| **Research team:** | Cristino Carneiro Oliveira  Diogo Carvalho Felício  Diogo Simões Fonseca  Anderson José  João Luiz Quagliotti Durigan  José Elias Filho  Túlio Medina Dutra de Oliveira |
| **Contact addresses:** | E-mail:carlamalaguti@gmail.com  **Tel.:**(32) 99199-3329 |
| **Unit/Department**  **/Institute/Institution:** | Faculdade de Fisioterapia/ Departamento de Fisioterapia Cardiorrespiratória e Musculoesquelética/ Universidade Federal de Juiz de Fora. |

**Drawing:**

This will be a randomized, prospective, clinical trial to be conducted in the Laboratory of Movement Analysis of the Federal University of Juiz de Fora School of Physical Therapy. Participants will be informed about the objectives of the research and those who agree to participate will be asked to sign the informed consent form. After approval from the research ethics committee, the trial will be registered in the Brazilian Clinical Trial Registries (REBEC) platform. Elderly subjects over 60 years of age from both sexes, will be eligible for the study. They will be examined in the geriatric ambulatory service of the university hospital of the Federal University of Juiz de Fora (HUCAS/UFJF). The subjects will be invited for participation by publicizing the study through posters or by invitation of the researcher in the waiting room, during their consultation visits to the ambulatory service. The evaluations will be initiated only after participants sign the Informed Consent Term. After checking for the eligibility criteria for inclusion in the study, the participants will be evaluated for the functional tests: walking speed, sit-up test, and the Timed Up and Go (TUG) test. Body composition will also be evaluated by analysis of the lean mass and fat mass using electrical bioimpedance. In addition, muscle strength during knee extension will be tested. Interviews will be conducted through questionnaires to enquire about their social participation and any fear of falling that they might have. After the 8-week intervention period, all parameters will be re-evaluated. The subjects will be followed up at 3 and 6 months, and re-evaluated through questionnaires about their fear of falling and to check their social participation. Participants will be randomly assigned to one of the two arms of the study, namely, electrostimulation group, or resistance training group. The electro-stimulation group will receive training based on whole-body electrostimulation (WB-EMS). Previous references that showed satisfactory results in the elderly with whole-body electrostimulation, will be used to define the electrostimulation parameters (Kemmler et al, 2014, 2013). The electro-stimulation group will be subjected to 18 minutes of electrical muscle stimulation, two sessions per week, for 8 weeks. The regimen will be as follows: an intermittent bipolar electric current with a frequency of 85 Hz, pulse width of 350 μs with 6 seconds of stimulation and 4 seconds of rest. The thigh, arm, abdomen, thorax, cervical and thoracic paravertebral muscles, and large muscles on the dorsal side will be stimulated. The **Resistance training group** will undergo two training sessions per week, with supervised resistance exercises involving the same set of muscles as those in the electro-stimulation group, but without electro-stimulation. They would be subjected to a load of 50 to 80% of one repetition maximum test (1-RM) for a period of 8 weeks. After the first reassessment, the subjects will be followed up at intervals of 3 and 6 months, and will be evaluated through questionnaires to check their social participation and fear of falling.

**Summary:**

The high prevalence of sarcopenia in the elderly and the role of exercise training in reversing it is well-known. However, there are several limitations that prevent the elderly from adhering to a regular physical training program to increase the muscle strength or mass and to reduce the risk of falls. Recently, whole-body electrostimulation has been reported to be effective for recovery from sarcopenia, with a short period of intervention. This study aims to evaluate the effectiveness of whole-body electrostimulation in improving the function, and muscle strength and mass in the elderly, as well as in reducing their fear of falling and increasing social participation, compared to conventional resistance training. The subjects will be elderly people, with no cognitive and neurological impairment, and no acute or chronic diseases or musculoskeletal ailments that prevent them from performing resistance exercise. The sample will be randomized to two groups. The electro-stimulation group will undergo 18 minutes of electrical stimulation of the muscles, two sessions a week, for eight weeks. The regimen will be an intermittent bipolar electric current with a frequency of 85 Hz, pulse width of 350 μs, with 6 seconds of stimulation and 4 seconds of rest. The resistance training group will perform exercises with a load of 50 to 80% of 1-RM in supervised sessions, twice a week, over a period of 8 weeks. Muscle strength will be evaluated by Microfet manual dynamometer; functional evaluation will be determined by gait speed, TUG test, and sit-up test; muscle mass will be evaluated by electrical bioimpedance, questionnaires will be used to evaluate the fear of falling and degree of social participation. The intergroup data will be compared by paired t-test or Mann-Whitney test according to the tested normality. Longitudinal evaluations after 3 and 6 months of training will be conducted through questionnaires, to evaluate the degree of social participation and the fear of falling. The effect size will be determined for each variable of interest. Analysis of mixed models, including the outcomes measured at the 4 time points (basal, after intervention, 3 and 6 months) will be performed. It is expected that the results of this study will determine the effectiveness of whole-body electrostimulation in improving the functional capacity of the elderly and in reducing the risk of falls.

**Keywords:**

Electrostimulation muscle, aging, sarcopenia.

**Introduction (literature review):**

An increase in the ageing population is a conspicuous worldwide phenomenon. In recent decades, developing countries have seen a progressive decline in their mortality rates and more recently, a decline in the fertility rates, leading to an increase in the elderly population. The definitive expression of this phenomenon can be seen in the population pyramid that is changing from a growing population model to a stabilized population model (Chaimowicz, 1997).

In Brazil, the socio-demographic data from the National Household Sample Survey (PNAD) of the Brazilian Institute of Geography and Statistics (IBGE, 2012) found that in 1991 the elderly represented 4.8% of the population, which increased to 5.8% in 2000, and 7.4% in 2010. Of the total Brazilian population of 190,755,799, the number of those aged 65 years or above is 14,081,480. The Brazilian Institute of Geography and Statistics (IBGE) estimate for the year 2025 is that the elderly are likely to comprise 15% of the total population, corresponding to approximately 30 million elderly in the country (IBGE, 2012). According to the World Health Organization (WHO 2005), by 2025 Brazil will have the sixth largest number of elderly people in the world.

In the country, medical advances and improvements in the general living conditions of the population have increased the life expectancy from 45.5 years in 1940 to 72.7 years in 2008, thus adding 27.2 years to the life expectancy. According to IBGE projections, the average life of its population will continue to increase, reaching 81.29 years in 2050. This is equal to the life expectancy seen today in some countries: Iceland (81.80), China (82.20), and Japan (82.60) (IBGE, 2012). Aging is accompanied by an increase in the prevalence of chronic degenerative diseases and comorbidities, leading to a decrease in the functional capacity, quality of life, and increase in dependence on others (Busscheet *al.*, 2011). Among the changes that occur in the aging process, sarcopenia is significant.

In 1989, Rosenberg proposed the term sarcopenia (from Greek, *sark=* meat; *penia=* loss) to describe the involuntary loss of age-related skeletal muscle mass. Some researchers reported that in addition to the loss of muscle mass, there is also a decrease in muscle strength (Sayeret *al,* 2008; Langet *al*, 2010). Other authors, defined sarcopenia as loss of skeletal muscle mass, muscle strength, and functional limitation (Evans, 1995; Roubenoff, 2001). In 2010, in an attempt to develop an operational definition of sarcopenia, Cruz-Jentoft et *al* proposed through a European consensus that sarcopenia is a geriatric syndrome characterized by loss of muscle mass and muscle function (strength or physical performance), without the need for disease for its appearance, although the process might be accelerated due to some chronic diseases. According to the consensus, muscle mass and strength can be detected in clinical practice by physical examination such as anthropometry and grip muscle strength, and in scientific research with more reliable instruments such as computed tomography and isokinetic dynamometry.

After thirty years of age, the muscle mass decreases by approximately 3-8% and this rate of decline is more pronounced after sixty years (Meltonet *al*, 2000). Baumgartner *et al* in 1998 conducted an epidemiological study to estimate the prevalence of sarcopenia among 883 elderly people in New Mexico. They found an increase in the prevalence of sarcopenia from 13%-24% in those aged above seventy, to 50% in those aged above eighty years. Sarcopenia was significantly associated with physical inactivity, morbidity, obesity, lower income, and change in health behaviors. In 2002, Iannuzzi-Sucich conducted a similar study among 195 women aged 64 to 93 years and found the prevalence of sarcopenia to be 22.6%. The prevalence was 26.8% among 142 men aged 64 to 92 years. Analyzing women and men above 80 years of age, the authors observed that the prevalence increased to 31.0% and 52.9% respectively. Multiple interrelated factors contribute to the development and progression of sarcopenia. It is postulated that with aging, there is a decrease in the level or resistance to anabolic substances in the skeletal muscle, such as testosterone and androgen. In addition, there is atrophy of type II fibers, decline in motor units, loss of alpha motoneurons, reduced protein intake, reduced growth hormone, sedentary lifestyle, and immunosenescence (Roubenoffet *al,* 2000).

Resistance exercise is one of the therapeutic modalities for the prevention and treatment of sarcopenia (Kryger, 2007).

Kruger et al. evaluated 6000 elderly subjects and reported that only 11% were enrolled in resistance training programs. In another study, Sluijs et al. proposed a resistance exercise program for the elderly suffering from chronic pain, and reported that 70% did not show satisfactory long-term compliance. Taylor et al. added that adherence to exercise among the elderly is approximately 14 to 17%. The term adherence refers to the number of sessions held, divided by the number of sessions offered (Hong SY et al, 2008). Data in the literature suggests that the results of an intervention in the elderly are satisfactory when adherence to the program is around 80-85% (Pistersetal, 2010).

Health professionals should make efforts to identify factors impacting adherence to resistance exercises. The elderly tend to participate more regularly at the beginning of the program and the absenteeism increases over time (Pisterset al, 2010). New training strategies might be able to optimize the adherence of the elderly to therapeutic programs. Recently, training with whole-body electrostimulation has been used as an alternative.

Whole-body electrostimulation has been suggested for muscle conditioning in recent times. It is a safe and non-invasive technique using the Mihabodytec® equipment (Gersthofen, Germany) that allows the simultaneous activation of several muscle groups. One of the advantages of WB-EMS is that it acts directly on the synthesis of musculoskeletal protein and demands much lower time than the conventional techniques.

Studies involving the elderly subjects are increasing. In a recent survey, Kemmler et al. (2014) conducted a randomized clinical trial that aimed to evaluate the effectiveness of WB-EMS in changing the body composition of elderly people at the risk of sarcopenia. Seventy-six elderly subjects were allocated to two groups. The experimental group underwent 18 minutes of electro-stimulation, three sessions every 14 days totaling 54 days of intervention. At the end of the program the researchers observed significant results in the experimental group in terms of appendicular muscle mass, lean mass, and in the strength of knee and trunk extenders.

In another study, the effectiveness of WB-EMS in increasing the appendicular muscle mass and abdominal fat in the elderly was investigated. Forty-six elderly subjects with a sedentary lifestyle were allocated to an experimental group (EG) and control group (CG). The EG (n=23) underwent 18 minutes of WB-EMS, three sessions every 14 days totaling 54 days of intervention. After 12 months of intervention, there were statistically significant results in favor of EG in the outcomes of appendicular muscle mass and abdominal fat (Kemmler et al 2013).

Despite promising results, there are few studies investigating the effectiveness of the WB-EMS . Moreover, cultural and socioeconomic influences can impact the results.

**Hypothesis:**

The main hypothesis of this study is that whole-body electrical stimulation is as effective as conventional resistance training in improving clinical-functional health outcomes.

**Primary objective**

The primary objective of this study is to compare the effect of whole-body electrostimulation and conventional resistance training on functional capacity, muscle strength, walking speed, risk of falling, and lean mass among elderly subjects.

**Secondary objective**

The secondary objectives of this study are to compare the effect of whole-body electrostimulation and conventional resistance training in reducing the fear of falling and increasing the social participation among elderly subjects.

**Procedures and Instruments:**

This will be a randomized, prospective, clinical trial to be conducted in the Laboratory of Movement Analysis of the Federal University of Juiz de Fora School of Physical Therapy. The subjects will be invited for participation by publicizing the study through posters at the university, personal communication, and dissemination through the internet and social networks. Participants will be informed and guided about the evaluations and objectives of the research, and those agreeing to participate will be asked to sign a Free and Informed Consent Terms. The evaluations will be initiated only after participants sign the Informed Consent Term. Participants will be instructed to wear comfortable clothing and appropriate footwear, and will be advised not to perform strenuous exercises at least 24 hours before the assessment and the exercise sessions. The allocation to the experimental and control groups after the baseline evaluation will be at random, using a table of random numbers.

All participants will be subjected to the evaluations described below, at baseline, immediately after the intervention, and eight weeks after the intervention. Questionnaires to evaluate the fear of falling and degree of social participation will also be administered at the same time points. The baseline assessment as well as re-evaluation after eight weeks of intervention will last approximately 40 minutes each. After the first re-evaluation, the subjects will be monitored at intervals of 3 and 6 months through the questionnaires about degree of social participation and fear of falling. These will be done through phone calls and will assess whether the effect of the training has been maintained over a year. The call for answering the questionnaire will last a maximum of 15 minutes, which will be the average response time for both questionnaires (Table 2).

Screening: The Screening Instrument (ANNEX 1) will be used to collect the data about demographic (e.g. age, gender) and clinical characteristics (medications, co-morbidities, clinical history, and family history).

The Six-Item Screener (SIS) (Annex II) is a simple tool to identify cognitive impairment. It was developed by Callahanet al. (2001), derived from the *Mini-Mental State Examination.* It can be administered over the phone or in the form of an interview. Its score ranging between zero and six, is easily marked by a simple sum of errors and consists of parameters to check the temporal orientation (three items) and late memory (three items). SIS will be administered soon after the Screening Instrument and patients who score less than four will be considered as having cognitive impairment and will be excluded from the study.

**Functional physical evaluation**

The **usual walking speed** will be assessed (Annex III). The distance to time ratio (m/s), measured over a space of 10 meters, will be used to evaluate the usual walking speed. Participants will be instructed to walk at a self-selected speed. The walking speed will be recorded only in the central 6 meters of the track identified laterally by tape marks, to avoid acceleration and deceleration bias. Participants will be instructed to stand with both feet behind the start line and start walking after the verbal command "*walk at your normal pace until the last mark on the ground, that is, as if you were walking on the street to make a purchase at the bakery"*; (Fritz 2009).

Another functional evaluation is the **Timed Up &Go (TUG**) test, which will be conducted according to the recommendations (Podsiadlo et al. 2009) (Annex IV). Participants will be seated in an armchair and will be asked to stand and walk for three meters, turn a cone, walk back, and sit on the chair. Participants will be encouraged to complete this walk as quickly as possible without running. The outcome will be the time taken to complete the task (Podsiadlo et al. 2009).

Additionally, the test will be performed using a chair without arms, with a height of 43.2 cm (Annex V). The chair will be placed against the wall to prevent displacement during the test. The test will begin with the participant sitting in the middle of the chair, back straight, feet approximately shoulder wide and resting on the floor at an angle slightly behind the knees, with one foot slightly in front of the other to help maintain balance when standing. The arms will be crossed against the chest. On receiving the "go" command, the participant will be asked to stand up (body upright and straight) and then return to the original sitting position. Participants will be encouraged to perform as many complete sit-up cycles as possible within a time limit of 30 seconds (Jones et al. 1999).

**Evaluation of strength of extensors of the knee:** Strength of the quadriceps muscles will be evaluated during the left and right knee extension, using the Microfet manual dynamometer (DMM) by means of isometric tests (Annex VI). The DMM is a reliable and valid assessment tool for measuring the muscle strength during knee extension in the elderly (Arnold 2010). With the participant sitting on a high chair, with the feet not touching the ground, the legs in a vertical position, and the dynamometer applied perpendicular to the leg proximal to the malleolus (Figure 1) and fixed by a strap to the chair, and the determination of the angulation of knee flexion will be performed with goniometer. The participant must push to extend the knee against the manual dynamometer. During the evaluation the verbal command will be given as "*stretch the knee by using greater force, stretch, stretch"*. This measure will be performed five times, and the highest and lowest force values recorded will be discarded. The average of the three remaining values will be calculated, and the isometric strength of the knee extensor muscles will be recorded in Newtons.


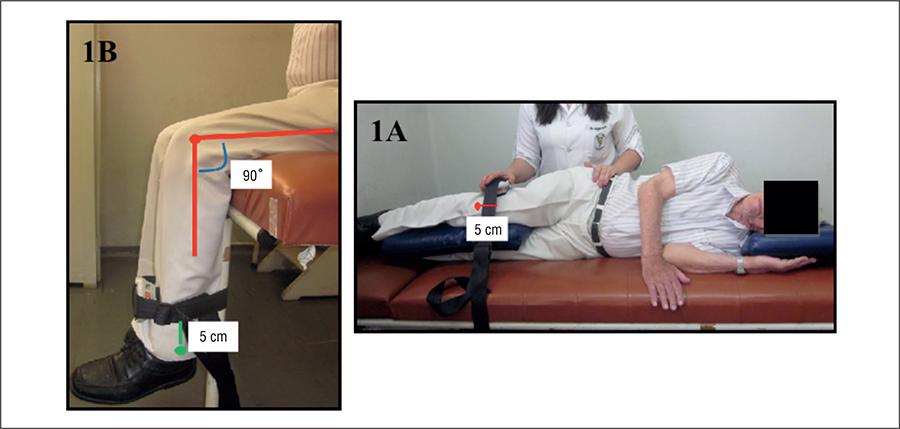


Figure 1. Patient positioning for evaluation of the extension force of the knee.

**Evaluation of muscle mass:** The body composition will be measured using the electrical bioimpedance apparatus *Biodynamics* tetrapolar, commonly described in the literature, which is a digital instrument, providing the values of resistance and reactance. The examination is performed by passing a low electric current (500 to 800 μA and 50 kHz) through the participant’s body, this imperceptible to the participant (Annex VII).

Compliance to the prior guidance given to participants will be verified before the examination. The guidelines require the patient to come to the test site on an empty stomach and not engage in physical activity until 12 hours before the test, not to consume alcohol for 48 hours before the test, and to empty the bladder 30 minutes before the test. The bioimpedance measurements will be obtained from the right side with the patient in a supine (lying down) position after removing the shoes and socks. The patient will be instructed to abduct the upper and lower limbs away from the body at approximately 30°, from the midline. The sites that will have contact with the electrodes will be cleaned with alcohol to ensure better contact with the electrode. The electrodes will be discarded after each measurement. The black cables (injectors) will be connected to the foot electrodes and the red cables (receivers) to the hand electrodes. “Alligator" connection clamps will be connected to the distal electrodes on the middle finger of the hand or near the toes. The red clamps will be placed on the proximal electrodes on the wrist and ankle. The detection electrode (red forceps) will be placed on the wrist on an imaginary line that divides the ulna and starts at the bony protuberance of the wrist; the black forceps electrode will be placed on the middle finger. On the foot, the electrodes will be placed on an imaginary line that divides the medial and lateral malleoli using the red forceps. The black forceps will be placed above the metatarsals of the toes (Figure 2).

The impedance, as opposed to the flow of running water, will be then measured. Before the examination, it will be checked whether the guidelines provided at the time of the interview have been complied with. Measures will be taken in the supine position after 15 minutes of rest. Using the resistance and reactance values obtained, the lean body mass (LBM) can be calculated using the equation: (LBM = total body water/0.732; being: total body water = 0.69 x height^2^ x resistance + 0.8). The lean BODYmass (kg) will be used to calculate the lean mass index (LMI), which is similar to the BMI, but uses the lean body mass (in kilogram)/height^2^ (in meters), to determine the muscle depletion (Pichard et *al*., 2000).

Figura 2. Illustration of the positioning of the electrodes during the bioimpedance evaluation.

**Evaluation of body mass and height:** The height of the subject expressed in centimeters (cm), will be measured in the orthostatic position after a deep inspiration, with both feet together and the weight of the body distributed on both feet. Stadiometer will be used to measure the height, and the values will be approximated to 0.5 cm. The body mass will be measured on a *Welmy®* mechanical anthropometric scale. This will be performed with the patients being barefoot and wearing light clothes, and will be expressed in kilogram (Kg).

**Social participation** will be assessed by the *Assessment of Life Habits* (*LIFE-H*) scale, which is divided into two sub-scales ("Daily Activities" and "Social Roles") that include twelve areas related to nutrition, fitness, personal care, communication, housing and mobility, responsibilities, interpersonal relationships, community life, education, work, and recreation. These categories correspond to the chapters of activity and participation component of the International Classification of Functioning, Disability and Health (ICF). Each item is scored according to the level of achievement and the type of assistance required to perform each activity; the scores range from zero (total restriction on participation) to nine (maximum participation). The following formula is used to obtain the score for each domain: (Σ scores*10)/(number of applicable items*9), and for the total score, the average score of each domain is considered. Due to the irrelevance of the categories "employment" and "education" for most elderly subjects, these will not be considered for the analysis; thus, there will be 10 categories and 59 items.

**Fear of falling** will be assessed by the International Falls-Efficacy Scale-Brazil (FES-I-BRAZIL) (Annex IX). The FES-I-Brazil is an adapted and validated scale for the Brazilian population that has adequate intra and inter-evaluator reliability. This questionnaire will be administered by the researcher physiotherapist trained for this evaluation, and will be conducted through an interview in a private and reserved environment. It comprises questions about 16 varied daily activities, from simple activities such as bathing, to activities that require a greater degree of independence, such as participation in social activities (Camargos et al, 2007). Each item has a score ranging from 1 to 4 points. A score of ≥ 23 is considered to be associated with sporadic fall history and ≥ 31 points is associated with recurrent fall.

**Intervention:** After the baseline evaluation and randomization, the participants will be submitted to one of the two muscle training modalities (electro-stimulation or resistance) for a period of eight weeks, as described below. For each training modality (electro-stimulation or resistance) a maximum of two participants will be allowed to undergo simultaneous training. Regardless of the training modality, two slots per week will be offered to each participant: Tuesdays and Thursdays or Mondays and Wednesdays during the morning or afternoon periods.

**Electro-stimulation group:** The participants assigned to this group will be submitted to whole-body electro-stimulation, which can simultaneously activate 8 to 12 muscle groups (upper legs, upper arms, abdomen, thorax, lower back, upper back, and large dorsal) with different intensity adjustments. This training will be performed in the Physical-Functional Performance Laboratory of the Faculty of Physical Therapy, UFJF, with up to two participants per session simultaneously. The bipolar electric current by MihaBodytec® devices (Gersthofen, Germany), will be initially applied with the following parameters: 85 Hz frequency, pulse amplitude of 350 𝜇s intermittently, with 6 seconds of stimulation to perform the movement and 4 seconds of rest (Table 1). The current intensity can be individually decided and modified during the electro-stimulation session. The applied protocol will be intermittent low intensity/low amplitude movement, based on the parameters described in other studies (Kemmler et al, 2014, 2013). Participants will be subjected to and supervised for 18 minutes of full body electrostimulation training, twice a week, for 8 weeks. The training sessions will be monitored by a trained physiotherapist, and with visual and acoustic stimuli guided by videos that exactly mimic the movements of the protocol. Based on previous studies, the WB-EMS protocol will follow the commercial WB-EMS settings with low load and narrow range of motion. It will involve five basic movements ("central exercises") shown in Table 1; these will be combined and slightly modified to generate 12 dynamic exercises that will be performed without any additional charge in a standing position. The training with WB-EMS will be structured in one to two series of 6 to 8 repetitions. The amplitude, speed, and corresponding intensity generated by the movement will be low (i.e. semi squat: flexion of the leg <35°) to avoid harmful effects of the exercise itself, but will be adequate to help muscle activation. In addition, no progressive increase related to exercise will be applied during the study. After the adaptation period of 4 WB-EMS sessions, the intensity of the current will be adjusted individually according to the participants' tolerance. Considering the difficulty of accuracy in prescribing the intensity of electro-stimulation due to regional differences, participants will be guided to maintain a perceived "strong" effort rate (Scale between "5 to 7" from 0-10) (Borg, 1990) during contraction. Current intensity is the key element for positive effects compared to the conventional exercise programs. The corresponding current intensity for each region of the body will be saved on the equipment chip to generate a fast, reliable, and valid setting during subsequent WB-EMS sessions.

Table 1.Concomitant movements during WB-EMS

1. Semi squat (6 s down) with arm extension/weight lifting (6 s up) with arm bending

2. Semi squat (6 s down) with trunk flexion (abs)

3. Semi squat (6 s down) with pulleys/semi squat (6 s up) with lifting

4. Semi squat (6 s down) with arm abduction (back)/semi squat (6 s up) with arm abduction (breast)

5. Semi squat (6 s down) and with rowing exercise (6 s up)

**Resistance training group:** The participants in this group will receive physical training in the weight training room of the Physical Education Faculty of UFJF. Each training session will include a 10-minute warm-up, including a walk and movement of different body segments: arms, wrists, fingers, shoulders, legs, and ankles. The resistance training will involve eight weeks of weight training (front puller, knee extender, straight supine, pulley, etc.) involving all major muscle groups. Participants in this resistance training group will be asked to attend two sessions per week for a period of 50 to 60 minutes each. Individual adaptations to the training protocol will be made regularly, according to individual performance. To define the training intensity, a load of 1-RM will be estimated. 1-RM has been widely used as the gold standard for checking the muscle strength. For the quantification of 1-RM, repeated attempts to obtain loads similar to the 1-RM test will be made (three to five attempts) with the same interval between the attempts (two to five minutes). The highest load performed by the participant, well positioned and without movement compensation, will be defined as 1-RM. The intensity will be based on the number of possible repetitions (week 1-2: fifteen repetitions, week 2-6: nine repetitions, with an intensity of 50-60% at 1-RM in the first two weeks, and then with 70-80% at 1-RM. Participants will be continuously asked about effort perception through Borg's symptom perception scale. In case of extreme fatigue, the intensity might be reduced or the session might be interrupted. A training volume of three series per exercise and 4 seconds between repetitions with 60 seconds rest between the series will be offered, and the participants will be encouraged to maintain muscle contraction for 6 seconds. Up to two participants might be trained simultaneously per session. This training will be accompanied by a physiotherapist and an experienced physical educator trained for the study protocol.

The duration of sessions of the two interventions will be different (18 minutes for the electro-stimulation group *vs*. 50-60 minutes for the resistance training group). This is because the whole-body electro-stimulation training stimulates simultaneous muscle contraction of several muscle groups and is therefore time-effective compared to the resistance training that works separately for each muscle group selected.

Table 2. Description of the phases of the study.

| Phase 1  Baseline assessment  -Gait speed  - Timed up and go  - Sit to stand test  - Maximal isometric knee extension  - Body composition  - Falls efficacy (FES-I)  - Social participation (Life-H) | RANDOMIZATION | Phase 2  Intervention  (8 weeks)  Electrostimulation    X  Resistance training | Phase 3  Post-intervention  -Gait speed  - Timed up and go  - Sit to stand test  - Maximal isometric knee extension  - Body composition  - Falls efficacy (FES-I)  - Social participation (Life-H) | Phase 4  Follow-up (Month 3 and 6)  -  -  -  -  -    - Falls efficacy (FES-I)  - Social participation (Life-H) |
| --- | --- | --- | --- | --- |

**Inclusion criteria:**

The study will include a convenience sample of seniors of both sexes above 60 years of age, with a cognitive level that is adequate to understand the evaluations and interventions of the study. The subjects will be recruited from the geriatric outpatient clinic of the University Hospital of the Federal University of Juiz de Fora (HUCAS/UFJF), and will be invited through posters that will be put up to publicize the study, and/or by invitation of the researcher in the waiting room during their visits to the outpatient clinic.

**Exclusion criteria:**

Elderly people with cognitive changes detected on the basis of cutoff score < four on the Six Item Screener (ANNEX I) proposed by Callahan et al. (2001), presence of an inflammatory disease in the acute phase, acute cardiovascular and metabolic diseases, neoplasia active in the previous five years, use of anti-inflammatory drugs, presence of neurological sequelae, fractures or surgical osteosynthesis in the last six months, and severe visual and hearing difficulties will be the criteria for exclusion. These diseases or comorbidities will be researched from the patient's medical records at the geriatric outpatient clinic, where they will be followed up and investigated by a screening instrument.

**Risks:**

Tests such as gait speed when walking, standing up and sitting from a chair can increase the heart rate and arterial pressure, cause fatigue, and mild to moderate dyspnea; however, these are normal signs during submaximal effort. These signs will be carefully monitored to ensure they remain within safe limits; the examiner will discontinue the test if there is a risk to the participant. If the participant is not able to continue the test or in the face of any extreme discomfort, the participant will be allowed to ask for discontinuation of the test.

The group of seniors who will undergo resistance muscle training with weight lifting, might feel muscle fatigue at the end of the exercise series; however, this feeling will be continuously monitored by a trained and experienced physiotherapist, and interruption will be allowed if the fatigue is intense. During resistance training there might be a slight increase in blood pressure, heart rate, and respiratory rate, which are normal signs indicative of moderate physical exertion; however, these signs should return to baseline values after a few seconds of effort. In case of any other signal or symptom that represents a risk of any adverse effect, the training will be discontinued and the participant will be monitored and advised rest until his or her prompt recovery.

Electro-stimulation can cause a slight tingling sensation, but without much discomfort. In case of cramps or muscular pains this will be immediately interrupted. Thus, the risks are minimal. Participants will be assisted by experienced and trained professionals and researchers, to avoid any intercurrence associated with the study procedures. In the event that a participant does not achieve prompt recovery, Mobile Emergency Care Service (SAMU) may be triggered.

The risks related to the use of the research project's questionnaires include exposure to the routine, recalling some stressful events such as tiredness and fear of falling when performing some activities, or discomfort over the time spent in filling out the questionnaire. These are minimal, but potential stressors. In case of a feeling of undue stress, the participant will be allowed to stop filling out the instruments at any time and will also be allowed to exit from participating in the survey. The interviews for filling the questionnaires will be conducted in a private environment, with the researcher being present with the subject in an individualized room for data collection. Researchers also guarantee the confidentiality and anonymity of the participants. The data will be presented only in averages for each group. No individual data will be revealed.

**Benefits:**

This study will help to determine whether electro-stimulation exercise of the entire body is as effective as resistance exercise (of lifting weights). Thus, participation by volunteers in this research might help in selecting the choice of treatment to improve the strength, muscle mass, and physical capacity of elderly people with sarcopenia who have a high risk of falls and physical disability. The treatment can improve the quality of life for such people. The advantage of this study is that if the effectiveness of electro-stimulation over resistance training is proven, it saves the time required for the process of physical training, because electro-stimulation requires less time than resistance training. Thus, this form of training can provide greater tolerance and increase the adherence among the elderly sarcopenic patients.

**Data analysis methodology:**

**Statistical analysis**

SPSS version 20.0 will be used. The data will be presented as average and SD or median and interquartile. Statistical analysis by intention to treat will be performed using the unpaired t-test or Mann-Whitney test. The effect size will be determined for each variable of interest. Analysis of mixed models, including the outcomes measured at the 4 time points (basal, immediately after intervention, and at 3 and 6 months) will be performed. The "time point" will be included as a class variable. Group, time, and group * time interactions will be inserted. This will evaluate the sole effect of the intervention, since the difference from baseline values at each time point will be evaluated. A value of p = 0.05 will be assumed for all tests.

**Primary outcome:**

Changes in muscle strength of extensors of the knee, walking speed, number of repetitions in the sit-leave test, and change in the lean mass in kilograms.

**Secondary outcome:**

Changes in fear of falling assessed by FES-I/Brazil and social participation by Life-H.

**Sample Size:**

It is expected that 66 elderly people will be involved in the study, 33 in each arm. However, to understand the size of the effect, a pilot study with 10 subjects will initially be conducted to confirm our forecast based on the sample calculation.

**Will there be use of secondary data sources (medical records, radiographs, demographic data, etc.)?**

(X) Yes ( ) No

| **Inform the number of individuals approached personally, recruited, or who will undergo some form of intervention:**  **66** | | | **Number:**  **66** |
| --- | --- | --- | --- |
| **Groups into which research participants will be divided** | | | |
| **Group** | **No. of participants** | **Interventions to be carried out** | |
| Group  Electro-stimulation | 33 | Functional evaluation: walking speed, TUG test and sit-up test. Evaluation of the strength of extensor muscles of the knee. Evaluation of lean body mass. Degree of social participation and evaluation of fear of falling. Muscle training by electro-stimulation of the entire body. | |
| Group  Resisted Training | 33 | Functional evaluation: walking speed, TUG test, and sit-up test. Evaluation of the strength of extensor muscles of the knee. Evaluation of lean body mass. Degree of social participation and evaluation of fear of falling. Muscle training through resistance exercises. | |

**Execution Schedule:**

Data collection will begin only after the project has been approved by the CEP.

| **Stage Identification** | **Start (DD/MM/YYYYY)** | **Termination (DD/MM/YYYYY)** |
| --- | --- | --- |
| Training the team on the study procedures | 01/03/2021 | 01/04/2021 |
| Data Collection | 04/05/2021 | 09/05/2022 |
| Data analysis | 12/05/2022 | 17/10/2022 |
| Screening of participants | 02/04/2021 | 05/10/2021 |
| Writing the final report and manuscript | 19/01/2023 | 25/04/2023 |
| Description of results | 17/10/2022 | 25/01/2023 |

**Financial budget:**

Material to be purchased with the researcher's own resources:

The necessary research instrumentation will be acquired through the release of the project resources approved in the Universal Call for Proposals FAPEMIG APQ-03054-17:

| **Item identification** | **Quantity** | **Capital**  **Value in Reais (R$)** |
| --- | --- | --- |
| 01 | **Full body electrostimulator**  **Brand:** Mihabodytec® **Function:** Simultaneous activation of muscle groups | 47.000,00 |

Instrumentation available at the Physical-Functional Performance Evaluation Laboratory of the Physical Therapy School of the UFJF:

| **Item identification** | **Quantity** | **Capital**  **Value in Reais (R$)** |
| --- | --- | --- |
| 01 | **Manual Dynamometer**  **Brand:** MircrofetFunction**:** muscle strength evaluation | 3.000,00 |
| 01 | **Electrical Bioimpedance**  **QUANTUM II - Brand**: RJL Systems, Inc. (USA) **Function**: evaluation of body composition | 11.800,00 |

**Financing:**

The full body electro-stimulator required for the research will be acquired by releasing the resources of the project approved in the Universal Notice FAPEMIG APQ-03054-17. The other equipment necessary for the project has already been acquired by the researcher and is available in the Laboratory of Physical and Functional Performance Evaluation of the Faculty of Physical Therapy of UFJF. Other smaller requirements such as cartridge and paper for printing the protocols will be acquired with the researcher's own resources.

**Proposes waiving the TCLE:**

( ) Yes ( X ) No

**Will there be sample retention for biobank/biorretory storage?**

( ) Yes ( X ) No

**References:**

ARNOLD CM, WARKENTIN KD, CHILIBECK PD, MAGNUS CR. The reliability and validity of handheld dynamometry for the measurement of lower-extremity muscle strength in older adults. J Strength Cond Res 2010;24:815-24.

ASSUMPÇÃO FSN, FARIA-FORTINI I, BASÍLIO ML, MAGALHÃES LC, CARVALHO AC, TEIXEIRA-SALMELA LF. Adaptação transcultural do LIFE-H 3.1: um instrumento de avaliação da participação social. Cad Saúde Pub 2016: 32

BAUMGARTNER RN, KOEHLER KM, GALLAGHER D et al. Epidemiology of sarcopenia among the elderly in New Mexico. Am J Epidemiol 1998; 147:755-63.

BORG G. Psychophysical scaling with applications in physical work and the perception of exertion. Scand J WorkEnviron Health1990; 16 Suppl1:55-8.

BUSSCHE HVD, KOLLER D, KOLONKO T. et al. Which chronic diseases and disease combinations are specific to multimorbidity in the elderly? Results of a claims data based cross-sectional study in Germany. BMC Public Health 2011;11:1-9.

CAMARGOS FF, DIAS RC, DIAS JM, FREIRE MT. Cross-cultural adaptation and evaluation of the psychometric properties of the Falls Efficacy Scale-International Among Elderly Brazilians (FES-I-BRAZIL). Rev Bras Fisioter. 2010;14:237-43.

CHAIMOWICZ F. A saúde dos idosos brasileiros às vésperas do século XXI: problemas, projeções e alternativas. Rev Saúde Pública1997;31:184-200.

CRUZ-JENTOFT AJ, BAEYENS JP, BAUER JM et al. Sarcopenia: European consensus on definition and diagnosis. Age and Ageing, 2010:39; 412-23.

EVANS, WJ. What is sarcopenia? J Gerontol A BiolSci Med Sci 1995;50:50-55.

FRITZ S, LUSADDI M. White paper: walking speed: the sixth vital sign. J Geriatr Phys Ther2009; 32:2-5.

HONG SY, HUGHES S, PROHASKA T. Factors affecting exercise attendance and completion in sedentary older adults: a meta-analytic approach. J Phys Act Health. 2008;5:385–397.

IANNUZZI-SUCICH, M.; PRESTWOOD, K. M.; KENNY, A. M. Prevalence of sarcopenia and predictors of skeletal muscle mass in healthy, older men and women. J Gerontol A BiolSci Med Sci, 2002;57:772–77.

INSTITUTO BRASILEIRO DE GEOGRAFIA E ESTATÍSTICA (IBGE). Censo Demográfico. Ministério do Planejamento e Orçamento, https://agenciadenoticias.ibge.gov.br/agencia-noticias/2012-agencia-de-noticias/noticias/20980-numero-de-idosos-cresce-18-em-5-anos-e-ultrapassa-30-milhoes-em-2017

JONES CJ, RIKLI RE,BEAM WC. A 30-s Chair-Stand Test as a MeasureofLowerBodyStrength in Community-ResidingOlderAdults. ResearchQuarterly for Exerciseand Sport. 1999; 79:113-119.

KEMMLER W, BEBENEK M, ENGELKE K, VON STENGEL S. Impact of whole-body electromyostimulation on body composition in elderly women at risk for sarcopenia: the Training and ElectroStimulation Trial (TEST-III). Age (Dordr). 2014;36:395-406.

KEMMLER W, VON STENGEL S. Whole-body electromyostimulation as a means to impact muscle mass and abdominal body fat in lean, sedentary, older female adults: subanalysis of the TEST-III trial. ClinInterv Aging. 2013;8:1353-64.

KRUGER J, BROWN DR, GALUSKA DA, BUCHNER D. Strength training among adults aged $65 years – United States, 2001. MMWR Morb Mortal Wkly Rep. 2004;53:25–28.

KRYGER AI, ANDERSEN JL. Resistance training in the oldest old: consequences for muscle strength, fiber types, fiber size, and MHC isoforms. Scand J Med Sci Sports 2007;17:422-30.

LANG T, STREEPER T, CAWTHON P,BALDWIN K, TAAFFE DR, HARRIS TB. Sarcopenia: etiology, clinical consequences, intervention and assessment. OsteoporosInt 2010;21:543-59.

NOREAU L, DESROSIERS J, ROBICHAUD L, FOUGEYROLLAS P, ROCHETTE AM, VOSCOGLIOSI C. Measuring social participation: reliability of the LIFE-H in older adults with disabilities. Disab and Rehab 2004; 26:346–352

OMS, Organização Mundial de Saúde. Envelhecimento ativo: uma política de saúde / OMS; tradução Suzana Gontijo. – Brasília: Organização Pan-Americana da Saúde, 2005.

PICHARD C, KYLE UG, BRACCO D, SLOSMAN DO, MORABIA A AND SCHUTZ Y. Reference values of fat-free and fat masses by bioelectrical impedance analysis in 3393 healthy subjects. Nutrition 2000; 16:245-54.

PISTERS MF, VEENHOF C, SCHELLEVIS FG, TWISK JW, DEKKER J, DE BAKKER DH. Exercise adherence improving long term patient outcome in patients with osteoarthritis of the hip and/or knee. Arthritis Care Res. 2010;62:1087–1094.

PODSIADLO D, RICHARDSON S. The timed "Up & Go": a test of basic functional mobility for frail elderly persons. J AmGeriatr Soc. 1991;39:142-8.

RAICHE M, HEBERT R, DUBOIS MF, GUEYE NDR, DUBUC N. Yearly transitions of disability profiles in older people living at home.EurGeriatr Med2012 55:399-405

ROUBENOFF R, CASTANEDA C. Sarcopenia: understanding the dynamics of aging muscle. J Am Med Assoc 2001;286:1230-1.

SAYER, A. A.; COOPER, C.; EVANS J. R. et al. The development origins of sarcopenia: using peripheral quantitative computed tomography to assess muscle size in older people. J Gerontol A BiolSci Med Sci, 2008;63:835-840.

SLUIJS EM, KOK GJ, VAN DER ZEE J. Correlates of exercise compliance in physical therapy. Phys Ther. 1993;73:771–782.

TAYLOR WC, SALLIS JF, LEES E, et al. Changing social and built environments to promote physical activity: Recommendations from low income, urban women. Journal of Physical Activity and Health. 2007;4:54–65.
